# Successful complex endovascular treatment of a contained rupture of a thoracoabdominal aortic aneurysm caused by *Mycobacterium bovis* after intravesical Bacillus Calmette-Guerin immunotherapy for bladder cancer

**DOI:** 10.1016/j.jvscit.2025.101895

**Published:** 2025-06-20

**Authors:** Laina Passos, Vaiva Dabravolskaite, Drosos Kotelis, Vladimir Makaloski, Michel Bosiers

**Affiliations:** Department of Vascular Surgery, Swiss Aortic Center Bern, University Hospital of Bern, University of Bern, Bern, Switzerland

**Keywords:** Ruptured suprarenal aortic aneurysm, Juxtarenal aortic aneurysm, Branched endovascular aneurysm repair, *M bovis*, Calmette-Guérin bacillus

## Abstract

A 71-year-old man underwent urgent endovascular repair using an inner branched endograft for a contained rupture of the visceral aorta. The patient initially improved, but later deteriorated. Positron emission tomography with computed tomography 9 months after surgery revealed perigraft inflammation. *Mycobacterium bovis* Bacillus Calmette-Guérin was identified from a hematoma puncture. He had undergone intravesical Bacillus Calmette-Guérin therapy 16 months earlier for bladder cancer. This led to an infected native aortic aneurysm. To our knowledge, this is the first reported case of infected native aortic aneurysm treated with an inner branched endograft. This case highlights the diagnostic challenges and suggests endovascular repair as a feasible option in selected high-risk patients.

Intravesical Bacillus Calmette-Guérin (BCG) therapy is a widely used and effective treatment for bladder cancer. However, it is associated with rare but severe complications, including symptomatic infective native aortic aneurysm (INAA). Since the first reported case in 1988, these events have been documented in the literature infrequently, with only 60 cases described between 1988 and 2022.^1^ Clinical manifestations are highly variable and often mimic inflammatory arteritis, and diagnostic imaging typically reveals features suggestive of an infectious etiology. We present the first reported case of a patient diagnosed with a contained rupture of the thoracoabdominal aorta that turns out to be an INAA caused by *Mycobacterium bovis* and linked to intravesical BCG therapy administered 16 months earlier for bladder cancer, which was treated successfully with an inner branched endovascular aneurysm repair (BEVAR). Written informed consent was obtained from the patient for the publication of this case report.

## Case report

**Fig 1:**
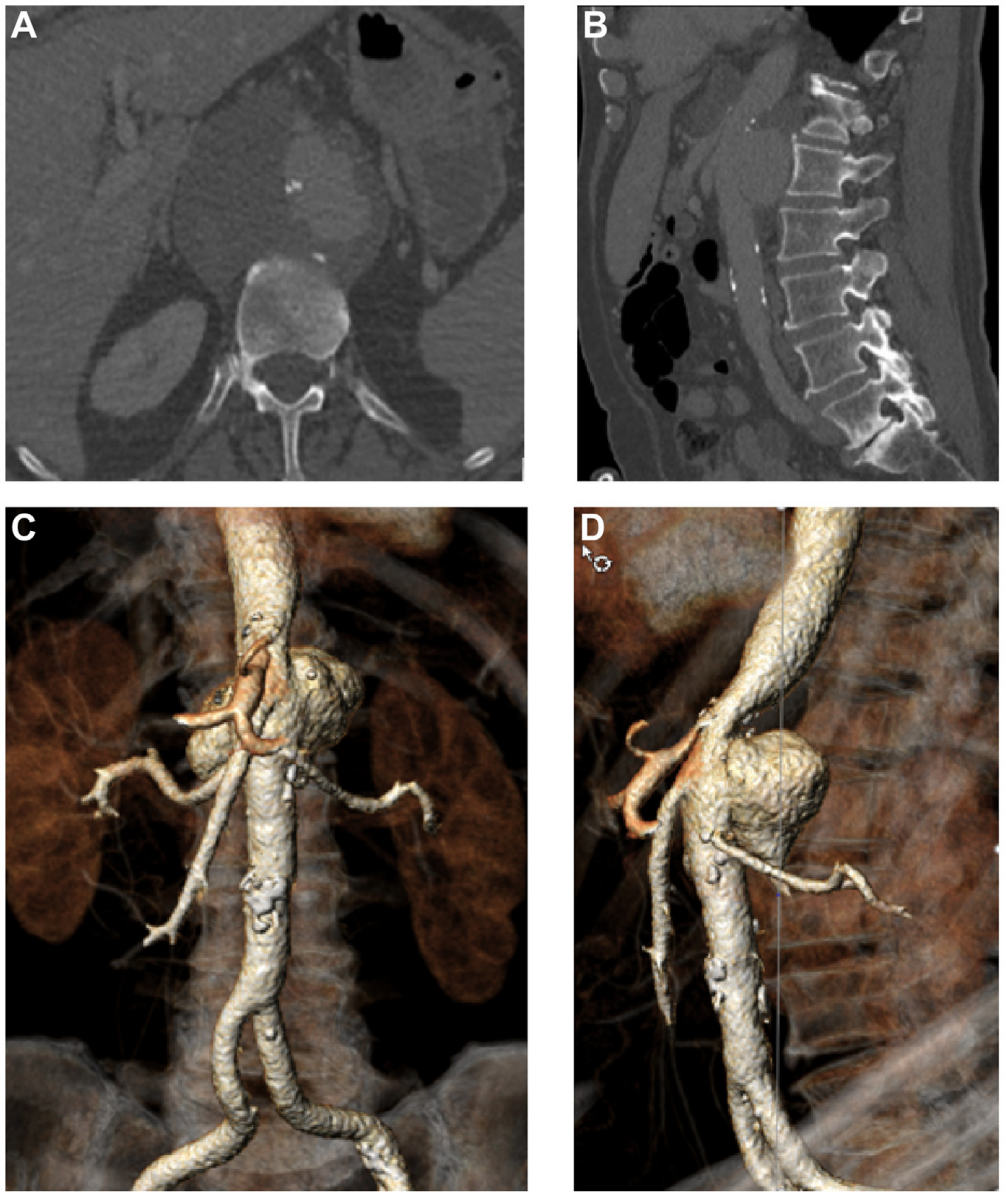
Preoperative contrast-enhanced computed tomography (CT) showing in an axial view **(A)** a rupture of a suprarenal aortic aneurysm and a retroperitoneal hematoma, and **(B)** in the sagittal view. **(C** and **D)** Corresponding three-dimensional reconstructions using *3mensio* software.

A 71-year-old man presented with sudden onset of abdominal pain that radiated to the back, accompanied by nausea and mild dyspnea. Contrast-enhanced computed tomography (CT) revealed a contained rupture of the thoracoabdominal aorta, characterized by an irregular aneurysmal wall, but without evidence of periaortic gas. Additionally, a retroperitoneal hematoma was identified (Fig 1). At this time, there were no definitive radiological signs suggestive of infection. Given the urgency, the anatomical location of the rupture, and the patient's comorbidities, an endovascular approach was chosen, despite the potential for an infective etiology. His medical history included coronary artery disease with elective stenting performed two years prior, as well as bladder carcinoma treated with transurethral resection of the bladder 6 and 22 years earlier, followed by intravesical BCG therapy that finished 16 months ago. Additionally, the patient had a history of chronic alcohol abuse (C2), active smoking, and arterial hypertension. Laboratory analysis revealed a leukocyte count of 10.2 × 10^9^/L and a C-reactive protein (CRP) level of 22 mg/L.

**Fig 2:**
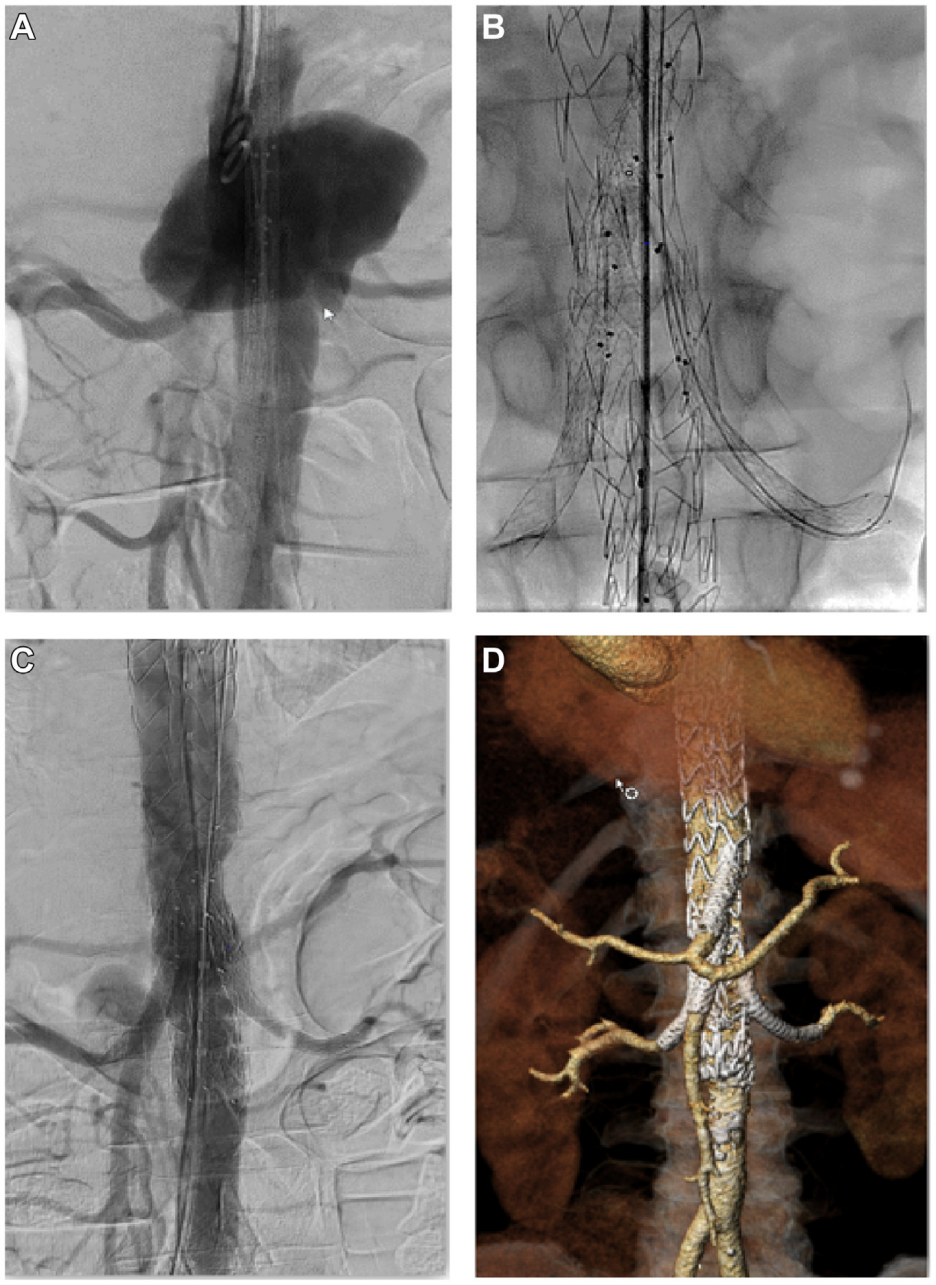
Postoperative angiography **(A-C)** showing proper positioning of the prosthesis. **(D)** Corresponding three-dimensional reconstruction using *3mensio* software.

The patient was urgently treated under local anesthesia. We successfully implanted an inner branched endograft (BEVAR) (Artivion E-Nside, JOTEC, Lotzenäcker, Germany) with attachment of the renovisceral vessels using Advanta V12 (Getinge, Sweden) with surgical access for the left axillary artery and percutaneous access for both common femoral arteries. He stayed in the intermediate care unit for 3 days, before being moved to the general ward. A postoperative CT revealed no complications (Fig 2). However, there was still a large retroperitoneal hematoma, for which we pursued conservative treatment, which included close clinical observation and regular radiological follow-up to monitor hematoma evolution and exclude signs of active bleeding or infection. Blood cultures remained negative.

During the postoperative phase, he tested positive for COVID-19 asymptomatically and developed mild leukopenia, presumably attributed to medication (metamizol), which had been documented previously in his medical history. Seven days after surgery, the patient was discharged home in good general condition. Blood analysis showed a CRP level of 47 mg/L and a leukocyte count of 2.94 × 10^9^/L. Lifelong aspirin therapy, clopidogrel for 6 months, and a statin were prescribed as part of the postoperative management. After discussion with the infectious disease team, antibiotic treatment was not initiated, given the absence of clinical and laboratory signs of infection.

At the 3-month follow-up, the patient reported exertional dyspnea and occasional palpitations, consistent with prior symptoms, without new complaints. CT confirmed proper endograft positioning, the absence of endoleak, decrease in aneurysm sac size, and regression of the retroperitoneal hematoma. Eight months after surgery, the patient presented with a deteriorating condition with weight loss, nausea, taste disturbances, evening chills, and nocturnal diaphoresis. Investigations revealed hepatosplenomegaly, bicytopenia (manifested as anemia and leukopenia), an elevated CRP (60 mg/L), and systemic inflammation markers. CT showed no evidence of tumor or infection. The cause of the inflammatory syndrome remained unclear; however, the clinical course was most suggestive of a reactive infectious process. Blood cultures remained negative. Although progressive splenomegaly could suggest a lymphoproliferative disorder, current imaging and laboratory findings did not support this hypothesis. Bone marrow aspiration revealed a low-grade B-cell neoplasm (5%-10% infiltration), consistent with chronic lymphocytic leukemia/small lymphocytic lymphoma. However, there were no clinical, laboratory, or imaging findings to support this entity as the primary cause of the patient's deterioration. Further hematological evaluation suggested monoclonal B-cell lymphocytosis, and the clinical picture was more consistent with a chronic inflammatory response. To further explore a potential infectious origin, screenings for HIV, hepatitis B virus, hepatitis C virus, and syphilis, as well as serological tests for cytomegalovirus, Epstein-Barr virus, brucellosis, and leishmaniasis were performed; all yielded negative results.

**Fig 3:**
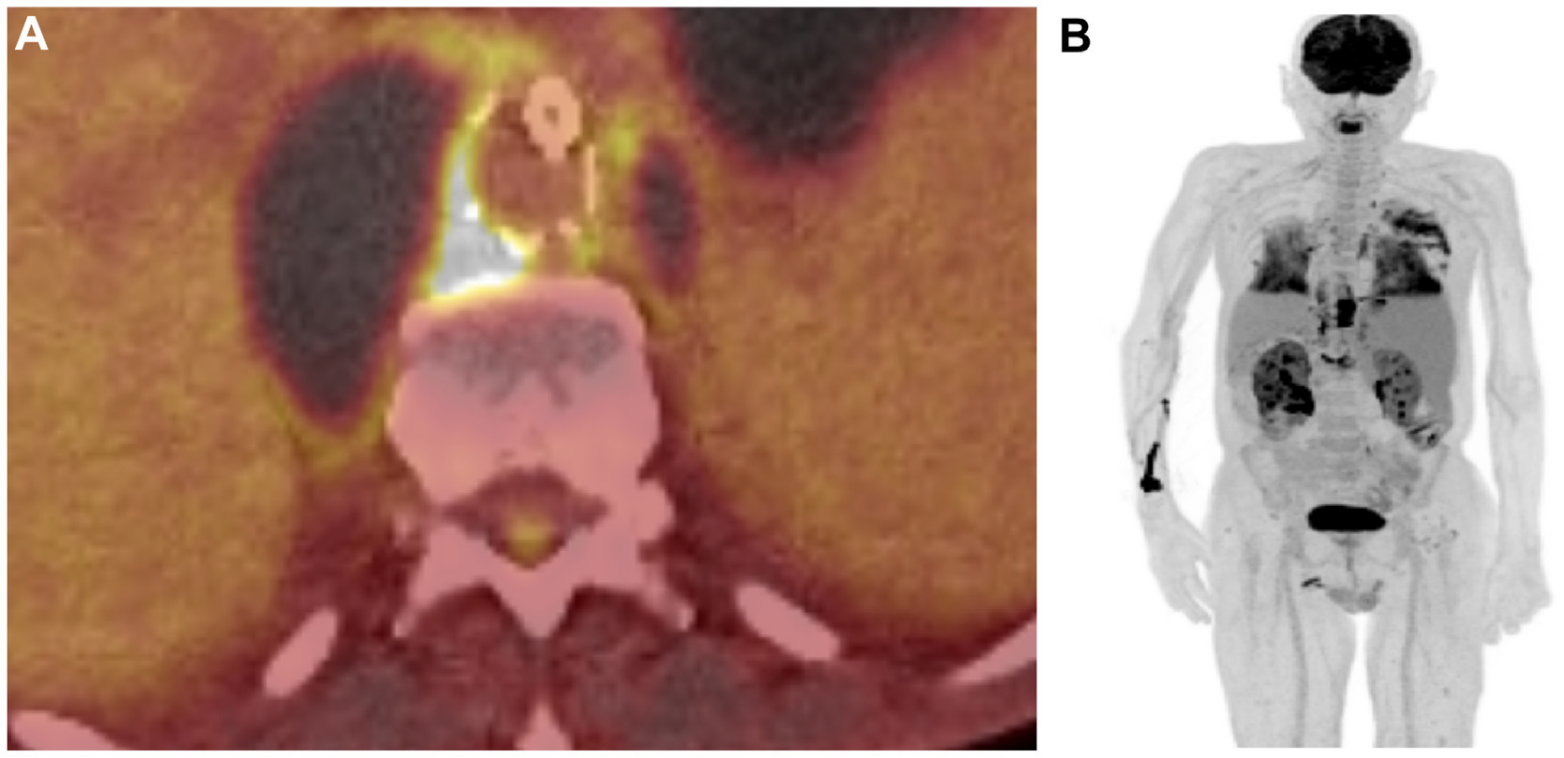
Positron emission tomography with computed tomography (CT) showing **(A)** an inflammatory focus around the thoracoabdominal graft and **(B)** in both lung lobes.

Acute renal failure was diagnosed during his hospitalization, with duplex ultrasound examination revealing bilateral renal artery bridging stent graft thrombosis. Percutaneous mechanical thrombectomy using Rotarex (BD, B. Braun, Melsungen, Germany), combined with drug-coated balloon angioplasty led to improvement in renal function. The patient still complained of dyspnea, fever, and decreased oxygen saturation. A positron emission tomography with CT 9 months after BEVAR identified inflammatory foci around the endograft and in both lung lobes (Fig 3), without evidence of disease progression. Serological tests for culture-negative pathogens yielded were negative. A biopsy of the hematoma was performed under radiological guidance, which revealed *M bovis*, confirming the diagnosis of an INAA.

The most likely transmission route of *M bovis* was intravesical BCG immunotherapy administered 16 months prior for bladder cancer treatment. The patient was initiated on a renal-adjusted triple therapy regimen consisting of rifampicin, isoniazid, and ethambutol for 9 months. His clinical condition showed gradual improvement, and after a 44-day hospitalization period, the patient was discharged to a rehabilitation clinic. The patient is still alive, 3 years after BEVAR.

## Discussion

Intravesical BCG instillations are used as a first-line treatment for superficial carcinoma of the bladder.^2^^,^^3^ Although usually well-tolerated,^2^ rare but serious complications such as INAA have been reported,^4^ linked to *M bovis*. Diagnosis is frequently delayed owing to the slow-growing nature of *M bovis* and the nonspecific clinical presentation, which can include low-grade fever or general malaise. CT remains the primary diagnostic tool; findings such as irregular aortic wall thickening, saccular aneurysms, and periaortic inflammation are not specific to this pathogen.^5^

The management of BCG-related INAA poses significant challenges. Definitive treatment traditionally involves open surgical debridement and reconstruction, often with biological grafts, followed by prolonged antimycobacterial therapy. However, open repair carries high mortality (varying from 18% to 43%),^6^^,^^7^ particularly in patients with comorbidities or anatomically complex aneurysms. Endovascular approaches offer a less invasive alternative, but are generally considered temporizing^2^^,^^8^ owing to the risk of persistent infection from unresected tissue.^9^

To our best knowledge, this case is the first reported use of an inner-branched BEVAR to treat a thoracoabdominal INAA caused by *M bovis* after BCG therapy. Previous reports at this anatomical level have used open repair exclusively. Our case demonstrates that, in select high-risk patients, complex endovascular reconstruction may achieve durable infection control when combined with prolonged antimycobacterial therapy—in this case, a 9-month course resulting in sustained remission. The decision to discontinue long-term suppressive therapy was based on the patient's favorable response to treatment, coupled with the ability to maintain close outpatient follow-up with regular clinical assessments and imaging surveillance.

This case highlights several educational points: the need for heightened clinical suspicion in patients with prior BCG exposure and atypical aortic symptoms, the diagnostic challenges posed by *M bovis*, and the potential for individualized, less invasive management strategies. As evidence evolves, such case reports may inform updated guidelines for managing vascular infections related to BCG, especially in anatomically difficult or high-risk surgical candidates.

## Conclusions

We report the first known case of an INAA owing to *M bovis* BCG infection after intravesical therapy that was treated successfully with an inner-branched BEVAR. These aneurysms may be under-recognized, posing significant diagnostic and therapeutic challenges. Management must be individualized and guided using a multidisciplinary approach.

## Funding

None.

## Disclosures

None.
